# Identifying factors that underpin student decisions to pursue the Doctor of Pharmacy degree at Atlantic Canadian Universities: Protocol for a mixed methods study

**DOI:** 10.1371/journal.pone.0316395

**Published:** 2025-01-22

**Authors:** Tiffany A. Lee, Amit Sundly, Stephen D. Coombs, Gerald J. Galway

**Affiliations:** 1 School of Pharmacy, Memorial University, St. John’s, NL, Canada; 2 Faculty of Medicine, Memorial University, St. John’s, NL, Canada; 3 Faculty of Education, Memorial University, St. John’s, NL, Canada; University of Science and Technology of Fujairah, YEMEN

## Abstract

Several international studies have investigated academic decision-making in higher education, but there is limited research on students’ choice to study pharmacy in the Canadian context. While there is some variation across jurisdictions, decisions to enroll in a particular degree program fall into several decision-making domains (e.g., personal, family, institutional, social, and economic). These findings have been theorized in various ways, for example, through social cognitive theory and social reproduction theory. The purpose of this study is to gain a better understanding of the personal, family, institutional, social, and economic factors that underpin student decisions to pursue the Doctor of Pharmacy (PharmD) degree at Atlantic Canadian Universities and to explore barriers to pursuing a pharmacy degree. The proposed study uses an explanatory sequential mixed-methods design consisting of a quantitative survey followed by qualitative interviews. All entry-to-practice PharmD students and graduates in Atlantic Canada are eligible to participate. The survey consists of several Likert scale questions associated with five decision-making domains, as well as several socio-demographic questions. Descriptive statistics and frequency counts will be used to describe the data; differences across decision-making domains, by gender and other demographic groupings, will be analyzed using inferential statistics. Semi-structured interviews with a sample of 12 to 15 participants will be conducted to further understand and explain the quantitative results. We will engage in thematic analysis of qualitative data. The findings of this research will provide insight into the decision-making patterns and socio-demographic characteristics of students who have chosen to pursue a PharmD. Important information will be gathered to inform health professional education and workforce planning, which we believe will contribute to improving healthcare resource capacity and patient outcomes in Atlantic Canada. The results of this project will also inform future recruitment strategies and admission criteria and support educators in the secondary school system in providing evidence-informed career counselling advice for students interested in pursuing a degree in pharmacy. The findings of this study may also be useful to educational leaders and policymakers.

## Introduction

### Evolution of pharmacist practice and education

Pharmacist scope of practice in Canada has evolved significantly over the past 10 years from a product-focused practice to a service-based, patient-centered practice whereby pharmacists have been afforded greater authority to prescribe, monitor, and administer medication therapy to meet the needs of their patients [1, 2]. As such, the role of pharmacists is expanding beyond medication dispensing to a broader scope of practice, including helping patients manage complex and diverse medical histories and navigating an increasingly complex and costly healthcare system [1]. Furthermore, pharmacists in Canada now provide public health services and patient-centered care activities, including immunization and point-of-care testing for communicable diseases and prescribing for common ailments [3]. Changes to models of care, national health systems, financial and economic shifts, and technological advancements have been postulated as the drivers of these changes [1, 4].

Changes in pharmacist scope of practice and models of care are associated with parallel changes in the curricula and pedagogy of post-secondary degree programs. In 2010, the Association of Faculties of Pharmacy of Canada (AFPC) and the Association of Deans of Pharmacy of Canada (ADPC) released a joint resolution and position statement intended to replace the baccalaureate pharmacy curricula with a comprehensive doctor of pharmacy curricula for the first professional degree in pharmacy [5]. Subsequently, AFPC merged its educational outcomes for baccalaureate and entry-level PharmD programs, presenting one set of educational outcomes for the First-Professional Degree Programs in Pharmacy with the overall goal of graduating medication therapy experts [6]. These efforts resulted in a pan-Canadian shift from a five-year baccalaureate to a six-year Doctor of Pharmacy degree [5, 7]. The Canadian Council for Accreditation of Pharmacy Programs (CCAPP) also announced that it will no longer accredit Bachelor of Science in Pharmacy programs beyond the year 2020.

The migration to the PharmD was accompanied by a sharp rise in tuition costs at most Canadian universities [8] and a conceptual shift in educational outcomes from graduating medication therapy experts to care providers with interdependent roles [9]. New accreditation standards, which come into effect in July 2024, also place emphasis on ensuring pharmacy graduates can work with and care for diverse populations, including Indigenous peoples and other equity-deserving groups (e.g., women, racialized persons, members of the 2SLGBTQIA+ community, persons with disabilities) and require universities to introduce mechanisms to recruit and admit persons who identify as members of these groups [10].

Compared to traditional approaches to pharmacy education and training in Canada, these changes (i.e., program length, curricular focus, tuition levels) represent significant shifts in how we prepare pharmacy students for practice and the financial resources required to earn a PharmD degree. These modifications lead to questions about how accessibility and participation in pharmacy programs might be impacted, particularly for traditionally underrepresented groups. Although there have been several international studies published which address academic and career decision-making in pharmacy education [11–17], similar research set in the Canadian context is limited. We could find no Canadian studies that examine the factors and influences that bear upon students’ decisions to study pharmacy. Nor could we locate research focusing on how recent changes in Canadian pharmacy programs are viewed by students and recent graduates. Although these are important program/policy questions, we do not yet know the factors and influences that drive students’ decisions to study pharmacy or whether recent program changes have led to accessibility issues.

The research project described here will fill some gaps in our understanding of the factors influencing pharmacy students’ decisions to study pharmacy in the Canadian context. It will also provide insight into pharmacy students’ socio-demographic profile and help identify barriers to accessing pharmacy education.

### Determinants and theoretical bases of academic decision-making

Several studies on academic decision-making have investigated factors within students’ social ecology that influence their pursuit of post-secondary studies in a particular field [18–23]. Sundly and Galway (2021) reviewed the literature on academic decision-making. They identified personal determinants (personal interest, academic performance, and career aspiration), institutional factors (career counselling, instructional focus, co-curricular involvement, and teacher beliefs), economic and occupational factors (income potential, the social prestige of a particular field, social mobility, labour market opportunities), social and cultural factors (prevailing beliefs, socioeconomic advantages, cultural background, and gender), and family factors (parental values, parental encouragement, parental education, parental income level, and family structure) as relevant to academic decision making [23]. Decisions to pursue a particular degree program have also been studied through several theoretical lenses, including rational choice theory [24], social cognitive theory [25], social reproduction theory [26], and social capital theory [27], among others.

While there is a general understanding of the factors associated with student’s decision to pursue post-secondary education [18–21], the diversity across academic fields in terms of economic value, job prospects, time commitment, and financial accessibility, among other factors, demand field-specific assessments of academic decision-making. Researchers within the Science, Technology, Engineering, and Mathematics (STEM) fields have realized such gaps and studied factors influencing students’ decision to pursue fields such as engineering [23, 28] and physical sciences [28].

With respect to pharmacy, several international studies have examined factors influencing students’ decision to pursue a degree in pharmacy. These include studies from the African [29–33] and Asian [11, 17, 34–36] countries, and anglosphere countries including Australia [37], New Zealand [13], the United States [12, 38], and the United Kingdom [14, 39]. The career goals of pharmacy students have also been explored in some studies [16, 32]. These studies used quantitative surveys, primarily consisting of demographic and Likert scale questions, to determine what influenced pharmacy students to pursue a career in pharmacy and how those influences varied based on different sociodemographic characteristics, such as race and gender. Factors that influence students to choose pharmacy as a career were identified as follows: good employment prospects and desire to work in the healthcare field [34, 37]; parental and teacher influence [30]; peer pressure, encouragement by pharmacists, work, volunteering experience, and attending career days [13]; and enjoying science subject matter, desire to work in health care and improving people’s health and well-being as well as rejection from medical schools [39]. However, the published literature in pharmacy education does not organize these determinants according to the five decision-making domains postulated by experts in higher education [23] or explore this concept using a qualitative or mixed methods approach. To our knowledge, this research represents the first Canadian study to explore academic decision-making in the field of pharmacy education.

In this protocol, we present the rationale for conducting our study, state our research objectives, discuss the methodology and methods we plan to use, and discuss the potential impacts of our research. The context for this study is Atlantic Canada, which has two comprehensive universities offering pharmacy education programs: Memorial University (Province of Newfoundland and Labrador) and Dalhousie University (Province of Nova Scotia). Our decision to situate the research within these programs is based on the nature and location of our practice and research network.

## Materials and methods

### Study aim and setting

This study aims to better understand the personal, family, institutional, social, economic, and sociodemographic factors that underpin student decisions to pursue the Doctor of Pharmacy degree at Atlantic Canadian Universities and explore barriers to pursuing a degree in this field. This study is being led by a multidisciplinary group of researchers at Memorial University in Newfoundland and Labrador. The objectives of this study are:

1. To identify factors influencing pharmacy students’ decision to pursue the PharmD degree.
2. To identify factors associated with students’ selection of the PharmD as their first choice of degree program.
3. To determine the extent to which influencing factors differ across decision-making domains (i.e. family, institutional, social, economic, and personal) by gender and other demographic groupings.
4. To explore, using in-depth interviews, how the identified factors enable and influence students’ pursuit of the PharmD degree and how these, or other factors, may also act as barriers, particularly in the context of equity-deserving groups.

### Study design

We will use an explanatory sequential mixed methods design consisting of a questionnaire/survey followed by qualitative interviews [40]. Consistent with the chosen mixed methods design, qualitative interviews will follow the quantitative data analysis [40]. The mixed method research (MMR) design is often utilized to study complex topics concerning human behavior. Furthermore, it is typically utilized in higher education research to study the complex nature of academic decision-making processes [41]. The quantitative phase will facilitate a comprehensive understanding of the determinants of students’ decision to pursue a PharmD degree, whereas the qualitative strand will enable further understanding and explanation of the quantitative results. In adopting this research framework, we are inherently assuming that the choice to pursue a degree in pharmacy is shaped by multiple factors, and these factors may differ from one individual to another. Therefore, a mixed-methods approach is warranted.

### Study participants

All undergraduate pharmacy students and graduates from the entry-to-practice PharmD programs at Memorial University and Dalhousie University who have provided informed consent are eligible to take part in this study. There are no exclusion criteria.

### Sampling and recruitment

A convenience sample of participants will be recruited to take part in the survey via email contact and advertisements shared on social media or posted on physical and digital message boards within the School of Pharmacy at Memorial University and Dalhousie University. Using the G*Power Analysis (version 3.1) for the t-statistic, the desired sample size for the quantitative survey was determined to be 278 participants based on the following assumptions: effect size 0.3; power 0.9; and alpha level 0.05 [42]. Survey participants will be asked to indicate their willingness to take part in a follow-up interview. Using a short demographic questionnaire, we will then select 12–15 participants to take part in an interview using purposeful sampling techniques, such as maximum variation sampling [43]. This approach will capture a broad range of perspectives to expand our understanding of the quantitative results.

Study advertisements will include the link or quick response code to the online survey. Individuals who consent to participate in the survey will be offered the opportunity to enter their name into a prize draw for one of five $25 gift cards. Similarly, all participants who take part in the interview will be offered a $20 gift card as a token of appreciation.

### Data collection and study duration

We will use the Qualtrics Survey Tool to administer the online survey. The survey instrument is a modified version of the questionnaire developed by Sundly and Galway [23] and Rosales and her co-investigators [28] for their research examining the decision-making of engineering students at Memorial University. The instrument was adapted for pharmacy students following a review of earlier studies in pharmacy education in other anglosphere countries with similar health care and educational systems, including Australia [37] and New Zealand [13], the United States [12, 16, 38], and the United Kingdom [14, 39]. The survey was then reviewed for validity by field-testing the instrument with a small group at Memorial University. Content validity (i.e., appropriateness, relevance, and representativeness of the questions to the measured construct) was established through consultation with pharmacy faculty members and subject experts at Memorial University and Dalhousie University who have experience taking part in pharmacy admissions and selection processes [44, 45]. Face validity (i.e., clarity, formatting, comprehensibility, and appropriateness for the target group) was assessed by the educational specialist (GG) on our research team as well as a group of students at Memorial University who are enrolled in the Doctor of Pharmacy for Working Professionals degree program (i.e., the post-graduate degree program for practicing pharmacists) [44, 45].

The finalized survey instrument consists of 61 questions. It is separated into three sections: (i) Likert scale questions assessing family, institutional, social, personal, and economic determinants of student’s decision to pursue a PharmD degree, (ii) Likert scale questions assessing the influence of change in the pharmacy degree program from the baccalaureate to the PharmD on students’ decision to pursue a pharmacy degree, and (iii) socio-demographic questions (e.g., age, gender, total household income) to enable the development of a socio-demographic profile of participants. The third section also asks participants to indicate whether the PharmD was their first choice of degree program. The Likert scale questions ask respondents to indicate the level of influence each factor had on their decision to pursue a pharmacy degree using a five-point scale from 1 (signifying ’not at all influential’) to 5 (signifying ’extremely influential’). Survey data collection began on March 27, 2024, and will continue until the end of the first week of August 2024 (i.e. one academic semester) or longer to ensure the target sample size is achieved.

Consistent with the explanatory sequential mixed methods design, the quantitative results will inform the development of a semi-structured interview guide and interview questions. Interviews will be conducted virtually using WebEx, a virtual meeting platform used by Memorial University. The interview questions will be centered on (1) probing key findings from the quantitative component of the research and (2) exploring how the identified factors enable and influence students’ pursuit of the PharmD degree and how these, or other factors, may also act as barriers, particularly in the context of equity-deserving groups (Objective 4). Interviews will be recorded and transcribed verbatim. NVivo qualitative research software will be used to assist with qualitative data analysis.

### Data management

Ownership of data resides with the first author (TL), with only the first (TL) and second (AS) authors having access to raw data. Data will be stored electronically as encrypted files and retained for a period of 5 years, after which all electronic files will be securely deleted.

### Data analysis

Quantitative data will be analyzed using the Statistical Package for Social Sciences (SPSS, version 29.0). Descriptive statistics will be used to summarize the data across the decision-making domains, gender, and other sociodemographic groupings. Data will be presented as frequency counts and percentages for categorical variables and as mean and standard deviation for continuous variables. This step provides an overview of the data distribution and an understanding of the interrelationship among the domains’ variables. For Objective 1, exploratory factor analysis (EFA) will be performed to identify factors that influence pharmacy students’ decision to pursue the PharmD degree with the internal consistency reliability reported using Cronbach’s alpha. This method assumes that the sample size is sufficient to ensure stable factor loadings. To evaluate whether the dataset is appropriate for EFA, we will use the Kaiser-Meyer-Olkin measure and assess the significance of Bartlett’s test. For Objective 2, logistic regression will be employed to explore the relationship between predictor variables (e.g., sociodemographic characteristics, decision-making factors) and the dichotomous outcome variable–students selecting the Doctor of Pharmacy as their first choice of degree program. For Objective 3, differences across decision-making domains by gender and other demographic groupings will be analyzed using inferential statistics. The choice of statistical test will depend on the characteristics of the data. For example, if the data is approximately normally distributed within groups and the variance is homogeneous across groups, parametric tests such as independent-samples t-tests (for two groups) or one-way ANOVA (for three or more groups) will be used. However, if the assumptions of normality or homogeneity of variances are violated, non-parametric alternatives such as the Mann-Whitney U test (for two groups) or Kruskal-Wallis test (for three or more groups) will be used. Statistical significance will be set at p<0.05 and effect sizes will be reported to provide a clearer interpretation of the practical relevance of observed differences.

Qualitative data will be analyzed using the thematic analysis approach described by Lichtman (2017), which involves reducing large qualitative data sets through iterative cycles of coding and re-coding and then moving from codes to categories and categories to concepts and themes [39]. This approach to thematic analysis is what Lichtman refers to as the “three Cs of analysis” [46]. NVivo will be used to organize data, codes, and categories. To ensure trustworthiness in the qualitative data analysis, we will engage two members of the research team in coding and categorizing data and all members of the research team in developing concepts and themes [40]. The development of themes will involve comparing, contrasting, and negotiating our interpretations of the data. We will consider our roles and positionality within the research by engaging in reflexive writing and team discussion [41]. This acknowledgement will ultimately enhance the thoroughness of data analysis and reporting.

### Ethical considerations

This research has been reviewed and approved by the Interdisciplinary Committee on Ethics in Human Research (ICEHR) at Memorial University (ICEHR number 20241411-PH) and acknowledged by the Health Sciences Ethics Committee at Dalhousie University with ICEHR serving as the Board of Record. Permission to recruit pharmacy students was also obtained from academic leadership at both faculties.

Informed consent will be obtained from all participants and documented accordingly prior to data collection. Participation in the survey is anonymous, and no personally identifiable information will be collected. The results of the quantitative survey will be presented in aggregate form only. Students who have consented to take part in an interview will be assigned a pseudonym and will not be identified in any documents, publications, or presentations. Only the first (TL) and second (AS) authors will know the identity of the interview participants. Other team members will have access to password-protected and de-identified transcripts to assist with interpretation of results.

## Discussion

The findings of this research will provide insight into the decision-making patterns and socio-demographic characteristics of students who have chosen to pursue a PharmD degree, including whether recent changes to PharmD curricula and pedagogy influenced their decision-making. This research will help to fill the gaps in scholarly research relating to accessibility issues in Canadian Pharmacy education for underrepresented and equity-deserving groups. We anticipate that the findings from our study will also be informative for health professional education and workforce planning, which may, in turn, contribute to improved health care resource capacity and patient outcomes.

A major strength of this study is the MMR design, which, to our knowledge, is the first to address the complexities of academic decision-making in Canada. Data collection involves the use of a survey instrument that has been pilot-tested and peer-reviewed. We expect the instrument to be adapted for use by researchers at other universities in Canada and beyond. From a policy and practice perspective, the knowledge gained from this research has the potential to inform admission policies and recruitment strategies at the university level. It may also be valuable to administrators and educators in the secondary school system—to help inform the development of evidence-informed academic advising and career counselling. Finally, we expect the findings from this research to support efforts to advance equity, diversity and inclusion within pharmacy practice and pharmacy education sectors and identify opportunities to create programs and initiatives to support equity-deserving groups through scholarships, financial and other supports at the school and university levels.

We also recognize certain limitations associated with this research. First, we acknowledge that respondents beyond their first year of studies are thinking retrospectively, and therefore, the decision-making process may be subject to recall bias. A longitudinal study could help determine whether similar or different results emerge as students advance through the PharmD program. Second, this study includes PharmD students and graduates from universities in Atlantic Canada and therefore, the results of the quantitative survey may not be generalizable to other universities in Canada.

We anticipate publishing the results of this study in scholarly journals and conference papers and presenting the results at academic conferences that will be open to members of the academic community. We will provide a summary of the research results to the faculties of pharmacy at Memorial University and Dalhousie University, as well as the respective faculties of education. We will also share the findings from this work with student organizations (e.g., the Canadian Association of Pharmacy Students and Interns) for their information.
